# Impact of Dietary Isoflavones in Standard Chow on Reproductive Development in Juvenile and Adult Female Mice with Different Metabolic Phenotypes

**DOI:** 10.3390/nu16162697

**Published:** 2024-08-14

**Authors:** Zianka Meyer, Sebastian T. Soukup, Anna Lubs, Daniela Ohde, Christina Walz, Jennifer Schoen, Holger S. Willenberg, Andreas Hoeflich, Julia Brenmoehl

**Affiliations:** 1 Working Group Endocrinology of Farm Animals, Research Institute for Farm Animal Biology (FBN), Wilhelm-Stahl-Allee 2, 18196 Dummerstorf, Germany; 2 Department of Safety and Quality of Fruit and Vegetables, Max Rubner-Institute, Federal Research Institute of Nutrition and Food, Haid-und-Neu-Straße 9, 76131 Karlsruhe, Germany; 3 Working Group Cell Physiology & Reproduction, Research Institute for Farm Animal Biology (FBN), Wilhelm-Stahl-Allee 2, 18196 Dummerstorf, Germany; 4 Reproduction Biology Department, Leibniz Institute for Zoo and Wildlife Research IZW, Alfred-Kowalke-Straße 17, 10315 Berlin, Germany; 5 Center for Internal Medicine, Section of Endocrinology and Metabolic Diseases, University Medicine Rostock, Ernst-Heydemann-Str. 6, 18057 Rostock, Germany

**Keywords:** isoflavones, diet, obesity, fertility, metabolism

## Abstract

Two factors influencing female reproduction have been repeatedly studied in different animal species and humans, namely, 1. secondary plant compounds, especially phytoestrogens (mainly isoflavones (IFs)), and 2. the physical constitution/metabolic phenotype (e.g., obesity). So far, these research results have only been considered separately. In this study, we investigated the influence on reproduction of both phytochemicals, mainly dietary IFs, and the metabolic phenotype represented by three mouse models considered as three distinct genetic groups (a control group, a mouse model with high metabolic activity, and a mouse line with obese body weight). The IF content in different investigated standard chows with similar macronutrient profiles varied significantly (*p* < 0.005), leading to high mean total plasma IF levels of up to 5.8 µmol/L in juvenile and 6.7 µmol/L in adult female mice. Reproductive performance was only slightly affected; only an IF dose-dependent effect on gestation length was observed in all genetic groups, as well as an effect on pregnancy rate in obese mice. Dietary IF exposure, however, caused earlier onset of vaginal opening by 4–10 days in juvenile mice (*p* < 0.05), dependent on the genetic group, resulting in a slight acceleration of sexual maturation in the already precocious obese model and to a strong earlier maturation in the otherwise late-maturing sporty model, bred for high treadmill performance. Therefore, our results may help to draw the missing line between the effect of dietary secondary plant constituents, such as IFs, and metabolic phenotype on sexual development.

## 1. Introduction

Isoflavones (IFs) belong to the group of phytoestrogens, which are naturally occurring non-steroidal secondary plant components found, for example, in red clover or soy. The quantitatively most relevant IFs in soy are daidzein, genistein, and, to a lesser extent, glycitein. Due to their abundance in soy, IFs play an important role in soy-based or soy protein-rich diets. Since livestock feed, whether concentrated or green fodder, can also be rich in phytoestrogens, consuming animal products can also lead to an intake of IFs, which is shown, for example, by varying amounts of daidzein, glycitein, and genistein in cow’s milk and dairy products as well as processed beef, chicken, lamb, and pork [1,2,3,4]. IFs can be metabolized by the gut microbiota, which alters their bioactivity [5,6]. For example, daidzein can be metabolized into equol, which exhibits a much higher estrogenic potency than its precursor, daidzein [7,8,9]. Equol can also be detected in different animal-based foods except butter, fish, and seafood [4].

Based on their structural similarity to 17-β-estradiol, phytoestrogen metabolites can display estrogenic or anti-estrogenic effects [10], mainly mediated by intracellular estrogen receptors (ERs) and estrogen-related receptors (ERRs). IFs can bind to both estrogen receptors (ER1 and ER2, also known as ERα and ERβ) with a higher binding affinity for ER2 [11]. Due to their competitive binding to ER, the estrogenic and anti-estrogenic effects depend on the coexisting estrogen concentration. IFs generally display a rather antagonistic effect at physiological estrogen levels, as their binding to ER is less potent than endogenous estrogen [10,11,12]. If, however, estrogen concentration is low, IFs can act as ER agonists [11,12], having a great influence on body composition [13,14,15], metabolism [16,17,18,19,20,21,22,23,24], and fertility [25]. Since the 1940s, with the advent of “clover disease” observed in sheep [26], it has become increasingly known that phytoestrogens can have endocrine effects on the synthesis, secretion, transport, metabolism, binding action, or excretion of natural hormones, not only in ruminants but also in rodents and potentially humans [27]. That may affect homeostasis, reproduction, development, and behavior and cause clinical manifestations. 

A recent review summarized the current knowledge of soy and soy IFs on women’s fertility, including the effects on hormonal status, menstrual cycle, and fertility [28]. In mice, several studies determined the impact of IFs on puberty onset [29,30], increased uterine weight [31], impaired fertility [32,33,34,35], increased pregnancy loss (with fewer implantation sites and increased resorption), prolonged estrous cycle without changes in serum estrogen [30,33], and upregulated estrogen-related receptor 1 (Esr1) but not Esr2 transcription in ovaries [36]. The outcome of the studies varies, demonstrating the impact of influencing factors such as the composition of the different IFs, dose, route of administration, age at administration, and, potentially, physical constitution. 

The connection between physical stature, particularly body weight, and human fertility has been repeatedly discussed. In recent studies, a negative effect of maternal obesity on reproduction was observed, in particular, delayed conceptions, increased rates of miscarriages, and a worsening of the metabolic and reproductive symptoms in fertility-related disorders [37,38]. Body weight, especially fat mass, was also shown to influence the age of puberty onset in both boys and girls [39,40]. In girls, the influence of body weight seems to have more of an impact on the age of puberty onset rather than the further course of puberty. Here, the term “critical body weight” or “critical fat mass” has gained importance, as it is thought that a minimum body weight/fat mass is necessary to enter puberty. The critical fat mass is described to be around 20% of total body weight [41]. While overweight or obese girls might reach this critical body weight earlier and, therefore, enter puberty at a younger age, underweight girls may show delayed puberty onset compared to their normal-weight peers [42]. Athletic girls and women characterized by decreased fat mass often show delayed puberty onset or menstrual irregularities [43,44,45]. The onset of puberty in humans is most commonly determined by the Tanner stage, which determines pubic hair and female breast development [46]. In mice, the onset of puberty is determined by the age at which vaginal opening (VO) occurs, usually around 26 days of age [47,48]. Similar to humans, the onset of puberty and other fertility-related parameters, such as pregnancy rate, can be influenced by body weight and fat mass [49,50]. 

For example, comparisons could be made between non-inbred Dummerstorf Control mice (DUC) and Dummerstorf mice selected for high body weight (DU6), in which a reduced litter size and increased litter weights were observed compared to the control [51]. Mice are characterized by fast reproductive development, which allows close investigation over several generations and the possibility of controlling environmental factors, such as food intake. As dietary intervention has gained attention in recent years to reduce the negative effects of obesity on female reproduction [52], phytoestrogens could represent one relevant component with potentially beneficial or adverse effects [27,38]. 

In our previous studies with the non-inbred mouse lines DUC and Dummerstorf mice selected for high Treadmill Performance (DUhTP), we observed differential effects of standard diets on body weight and fertility (e.g., litter size) in both lines, suggesting interactions between genotype and feed in mice [53]. 

Since these diets were produced from different soybean intermediates, we suspected different levels of IFs in both diets and hypothesized that the administration of diets with different IF content would have phenotype-dependent effects on body weight and fertility in our models, which may also be relevant to humans. To test this hypothesis, we conducted a study that combined three mouse lines initially derived from the same mouse strain but differing in body composition and metabolism due to long-term selection (representing three different genetic groups) [51] and three diets that differed in IF content. We used the two standard diets of the previous trial [53], which were assumed to have different IF levels, and a diet with a very low IF concentration (representing three diet groups) to investigate the effect on reproduction (occurrence of pregnancy, gestation period, number and weight of offspring) and sexual maturation and development (day of vaginal opening, ER expression in the ovaries).

## 2. Materials and Methods

### 2.1. Animals and Husbandry

All in vivo experiments were approved by the internal institutional review board from the Research Institute for Farm Animal Biology (FBN, Dummerstorf, Germany) and adhered to national and international animal protection guidelines (German Animal Welfare Act (TierSchG)). This study was conducted at the FBN Lab Animal Facility. We used the mouse lines DUhTP, generated by paternal selection over 140 generations for high treadmill performance, DU6, established by paternal selection for high body weight at day 42 for around 190 generations, and an unselected control mouse line (DUC). All lines, kept as non-inbred lines, were derived from the identical base population Fzt:DU, initially generated by cross-breeding of four inbred and four outbred mouse strains [51]. 

Although the DUhTP and DU6 lines originally (over 40 years ago) descended from the same line (Fzt:DU), represented by the unselected control DUC, all three mouse lines are diverse due to the selection procedures. Compared to the DUC, the DU6 line is characterized, on one hand, by a higher body weight (selection trait), but, on the other hand, also by generally larger body size and increased fat mass, particularly abdominal fat mass. The DUhTP line, in contrast to the DUC line, shows a higher running capacity on a computerized treadmill (selection trait) and exhibits major differences in metabolism, with a high dependence on lipids due to selection. The lines DU6 and DUhTP are both characterized by a lipid-based phenotype. However, the fat depots differ significantly, as the DUhTP line has more beige fat and thus more metabolically active tissue, while the DU6 mice accumulate white adipose tissue in an obese manner [51,54].

Animals were housed under specific-pathogen-free (SPF) conditions and kept in polysulfone cages of 267 × 207 × 140 mm^3^ (H-Temp PSU, Type II, Eurostandard Tecniplast, Hohenpeißenberg, Germany). Environmental conditions were defined by a 12 h light–12 h dark cycle (room temperature = 22.5 °C ± 0.2 °C, humidity = 40%–60%). The animals had free access to autoclaved pelleted food Ssniff^®^ M-Z food (Ssniff-Spezialdiäten GmbH, Soest, Germany), and water. Hygiene management and health monitoring in SPF husbandry were performed according to the recommendations of the FELASA.

### 2.2. Study Design and Sampling

**Figure 1:**
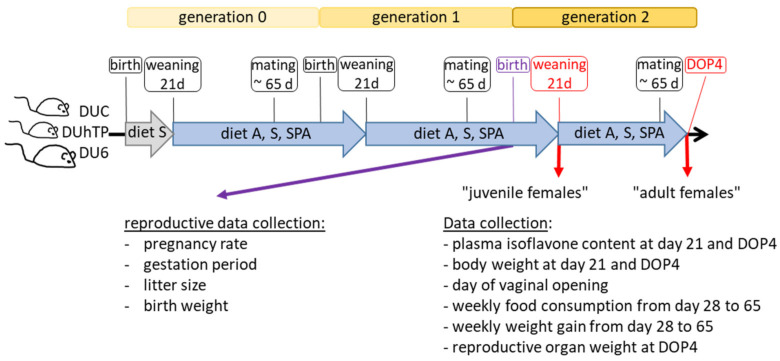
Experimental study design. On the day of weaning, males and females of the non-inbred lines DUC, DUhTP, and DU6 from genetically different litters/families were each divided into three groups and fed with unautoclaved diet A, autoclaved diet S, or diet SPA. Two mating periods were performed while avoiding inbreeding. The female offspring of the second mating, corresponding to generation 2, were used for the investigations on day 21 and day 4 of pregnancy (DOP4). Abbreviations: DUC—Dummerstorf control line, DUhTP—Dummerstorf mice paternally selected for high treadmill performance, DU6—Dummerstorf mice paternally selected for high body mass, A—Altromin, S—Ssniff^®^, and SPA—Ssniff phytoestrogen-poor alternative.

Animals from generation 145 (DUhTP), 180 (DU6), and 191 (DUC) were used for the experiment. On day 21 of age, animals from all lines were weaned, weighed, transferred to an isolated husbandry room under SPF conditions, and kept for two generations as a subpopulation without selection procedure but avoiding inbreeding (Figure 1). Mice in this parallel husbandry were separately kept in individually ventilated cages (GM500 Mouse IVC Green Line, Tecniplast, Germany). The animals received either diet A (#1314, Altromin, Lage, Germany, unautoclaved), diet S (Ssniff^®^ M-Z food, autoclaved), or diet SPA (Ssniff phytoestrogen-poor alternative, #V1154-300, Ssniff-Spezialdiäten GmbH, autoclaved) ad libitum. Diets A and S were used because they were the standard diets fed to the Dummerstorf long-term selection lines (diet A in the semi-barrier until 2010 and diet S in the SPF housing from 2010). A previous comparative study [53] already provided indications of possible different levels of IFs in these two diets. The IF-free diet (diet SPA) from the same company as diet S was the corresponding control diet. The selected standard feeds were treated according to the manufacturer’s instructions and corresponded to those used in the preliminary trial, in which the non-autoclavable feed for semi-barrier housing and the autoclavable alternative feed for SPF housing were used, and the different reactions to them were observed [53].

Animals were bred within their chow groups over two generations (generations 0–2, Figure 1). In every generation, litter size was standardized to ten pups per litter. The pups were weaned and separated at day 21 and mated around six weeks later, avoiding inbreeding. 

Only female animals were examined in this study. A total of 247 female mice (DUC_A_: n = 25, DUC_S_: n = 24, DUC_SPA_: n = 22, DUhTP_A_: n = 32, DUhTP_S_: n = 27, DUhTP_SPA_: n = 29, DU6_A_: n = 25, DU6_S_: n = 44, DU6_SPA_: n = 19) were included. While all 247 females were taken into account for the non-invasive observation of vaginal opening, fewer animals were used for the other analyses. The exact numbers are in the results section below the figures or within the tables. 

In particular, in generation 1, a maximum of 20 females per feeding group was bred (DUC (n ≥ 17), DUhTP (n ≥ 17), and DU6 (n ≥ 12)), and reproductive data, such as pregnancy rate, gestation period, litter size, and birth weight of ten offspring, were collected in generation F1 after mating. 

In the second generation, sexual maturity was determined by identifying the day of the vaginal opening, starting from day 15 (DU6, n ≥ 19 per feeding group) and 19 (DUC, n ≥ 22 and DUhTP, n ≥ 27 per feeding group). In addition, matings were carried out at slightly different times for each line to cope with the workload. The matings were started with the DU6 mice and then continued with the DUC and DUhTP mice (in the order in which puberty onset occurs). Successful mating was assumed by the presence of a vaginal plug, with this day dated as DOP 1 (Day of Pregnancy 1). On day 21, 72 females (n = 8 per diet group and line), and, on DOP 4, 107 females (DUC (n ≥ 11), DUhTP (n ≥ 13), and DU6 (n ≥ 8)) were sacrificed, and blood was either incubated at room temperature for 30–60 min and centrifuged twice at 5000× *g* (10 min and 5 min, respectively) to obtain serum samples or incubated with EDTA at 4 °C for at least 30 min and then centrifuged at 5000× *g* for 10 min to obtain plasma samples. The collected plasma and serum samples were stored at −20 °C. From adult females (DOP4), reproductive organs (uterus, ovaries, and fallopian tubes) were extracted, weighed (wet weight), snap-frozen in liquid nitrogen, and stored at −80 °C. 

In addition, the weekly feed intake of 10 animals per group (for DU6_A_: n = 8 mice) was recorded between the experimental days 28 and 65.

### 2.3. Quantitation of IFs in the Diets and Plasma Samples

The contents of the IFs daidzein, genistein, and glycitein, as well as their corresponding 6″-O-malonylglucosides, 6″-O-acetylglucosides, and glucosides in the diets, were determined by HPLC-DAD after extraction of the analytes as described previously [55] with a minor alteration: the single 6″-O-malonylglucosides and 6″-O-acetylglucosides were quantified by the respective glucoside reference standard daidzin, genistin, or glycitin. The values obtained this way for the 6″-O-malonylglucosides and 6″-O-acetylglucosides were corrected by individual correction factors. These correction factors were determined previously by measuring reference solutions of the 6″-O-malonylglucosides, 6″-O-acetylglucosides, and glucosides at the same concentrations and comparing the respective peak areas of the analytes. Three to four independent batches of each diet (diet A, diet S, and SPA) were analyzed. The values of each individual sample (batch) resulted from two independent analyses. The aglycone equivalents for the glycosides were calculated to determine the IF content of aglycone in the diets. Therefore, the different glycoside contents of the corresponding IF (daidzein, genistein, or glycitein) were converted into the respective aglycone contents based on their molecular weight. These single aglycone equivalents were summed up within one sample.

The plasma concentrations of daidzein, genistein, and their corresponding phase-II-metabolites, as well as equol, equol-7-glucuronide, and equol-4′-sulfate, were quantified after extraction of the analytes by UHPLC-MS/MS as described previously [56,57] with the following minor alteration: 50 µL instead of 100 µL study sample (mouse plasma) were used and diluted with 450 μL instead of 400 µL of water. 

For statistical analyses, IF concentrations between the limit of quantitation (LOQ) and limit of detection (LOD) were set as LOQ/2, and no detected analytes (<LOD) were set as zero. The LOQ and LOD are summarized in the Supplemental Material (Table S1).

### 2.4. RNA Isolation and Gene Expression Analysis

Ovaries of adult mice were homogenized using gentleMACS™ M Tubes (Miltenyi Biotec GmbH, Bergisch Gladbach, Germany), 350 μL Lysis-buffer (NucleoSpin^®^ RNA Kit, Macherey-Nagel, Düren, Germany), and the ‘RNA-program’ of the gentleMACS™ Dissociator (Miltenyi Biotec GmbH). Samples were centrifuged at 2500× *g* for 1 min, and supernatants were removed by pipette. RNA isolation was performed according to the manufacturer’s protocol using the NucleoSpin^®^ RNA Kit (Macherey-Nagel). Potential DNA contaminations were removed by a rDNase solution included in the kit. Extracted RNA was quantified using NanoDrop^TM^ (Thermo Scientific™, Waltham, MA, USA). Samples were stored at −70 °C until further analysis. Reverse transcription was performed in a 20 µL final reaction system consisting of 1 µg of total RNA, a mixture of random hexamers (2.5 μM) and oligo(dT) primers (2.5 μM), 10 mM dNTP-Mix, and RevertAid reverse transcriptase (200 U, Thermo Scientific^TM^). The level of mRNA expression was determined by quantitative real-time PCR (RT-qPCR) using LightCycler^®^ 96 instrumentation (Roche Diagnostics, Mannheim, Germany). For gene-specific amplification, cDNA samples were amplified in a 12 µL reaction mixture containing 0.5 μM each forward and reverse primers and 1× SensiFast SYBR No-ROX mix (Bioline GmbH: London, UK) according to the manufacturer’s instructions. The sequences of the primer used for amplifying Actb, Sdha, and Gapdh (housekeeping genes) and estrogen receptors 1 and 2 (Esr1 and Esr2) are shown in Table S2. After an initial denaturation (10 min, 90 °C), templates were amplified for 40 cycles consisting of denaturation (5 s, 95 °C), annealing (15 s, 60 °C), and elongation steps (10 s, 72 °C). A standard curve was generated for all assays by amplifying serial dilutions of a cDNA pool and subsequently analyzed using the LightCycler^®^ 96 software (Roche Diagnostics). The analysis yields an amplification efficiency of >90% for all primers (Table S2). Template normalization was performed with the software DAG (version 1.0.5.6) [58] using the housekeeping genes Sdha, Actb, and Gapdh. 

### 2.5. Quantification of 17 β-Estradiol by ELISA

The compound 17 β-estradiol was quantified in serum samples using a highly sensitive ELISA (ADI-901-174, Enzo Life Sciences Inc., Lörrach, Germany) with a 14 pg/mL detection sensitivity. Therefore, 30–50° µL serum was mixed with 400 µL cold mixture of acetone:acetonitrile:methanol (1:1:1), as performed previously [59], and was then sonicated for 15 min. After centrifugation (15 min, 4 °C, 14,000 rpm), the supernatant was dried using Speedvac (Eppendorf, Hamburg, Germany) and rehydrated in 125 µL assay buffer. Further procedures were performed according to the manufacturer’s instructions. The quantity of 17 β-estradiol in the samples was interpolated via the standards provided, according to the instructions. The correlation coefficient (R^2^) was calculated for each assay plate and varied between 0.9958 and 0.9979. Samples with a calculated 17 β-estradiol level below 20 pg/mL were considered “below the detection limit” and excluded from subsequent calculation.

### 2.6. Statistics

The statistical analyses were performed using GraphPad (GraphPad Prism 9.5.1; San Diego, CA, USA). Outliers were identified and excluded using the ROUT method (Q = 0.2%). All data were analyzed using two-way ANOVA with multiple comparisons, considering the three different mouse strains (DUhTP, DUC, DU6) and the three different diets (A, S, SPA). One-way ANOVA was applied by comparing diet A and diet S regarding the content of daidzein, genistein, glycitein, and overall sum in aglycone equivalents. The same test was used to determine line-specific or diet-mediated effects on body weight, Esr1 as well as Esr2 expression, and estradiol concentration. The two-tailed Chi-Square Test was performed to evaluate pregnancy data. Pearson correlation coefficients were calculated to determine the impact of plasma IF concentration on Esr1 and Esr2 mRNA expression and the relative uterine and ovary weight within the respective mouse lines DUC, DUhTP, and DU6. The effects and differences between groups were considered significant when *p* < 0.05.

## 3. Results

### 3.1. IF Profile in Three Different Commercial Mouse Diets

The IF profile in the feeds was measured by HPLC-DAD. The aglycone equivalents for the glycosides were calculated to determine the IF content as aglycone in the diets (see Section 2.3). Samples from independent batches were analyzed for each feed. In the SPA feed, no IFs were detected (Table 1). Diet A had a significantly higher content of IFs (overall IF aglycone equivalents: 0.594 mg/g) than administered diet S (0.392 mg/g; *p* < 0.05). The content (as aglycone equivalents) of total daidzein (diet A: 0.267 mg/g, S: 0.157 mg/g), total genistein (diet A: 0.287 mg/g, diet S: 0.195 mg/g), and total glycitein (diet A: 0.041 mg/g vs. diet S: 0.039 mg/g), instead, did not differ significantly between the chows. Nevertheless, in percentage terms, diet A had significantly more daidzein in aglycone equivalents (45.1%) than diet S (39.9%, *p* < 0.005).

### 3.2. Effect of Different Diets on Reproduction of the 1st Generation

**Figure 2:**
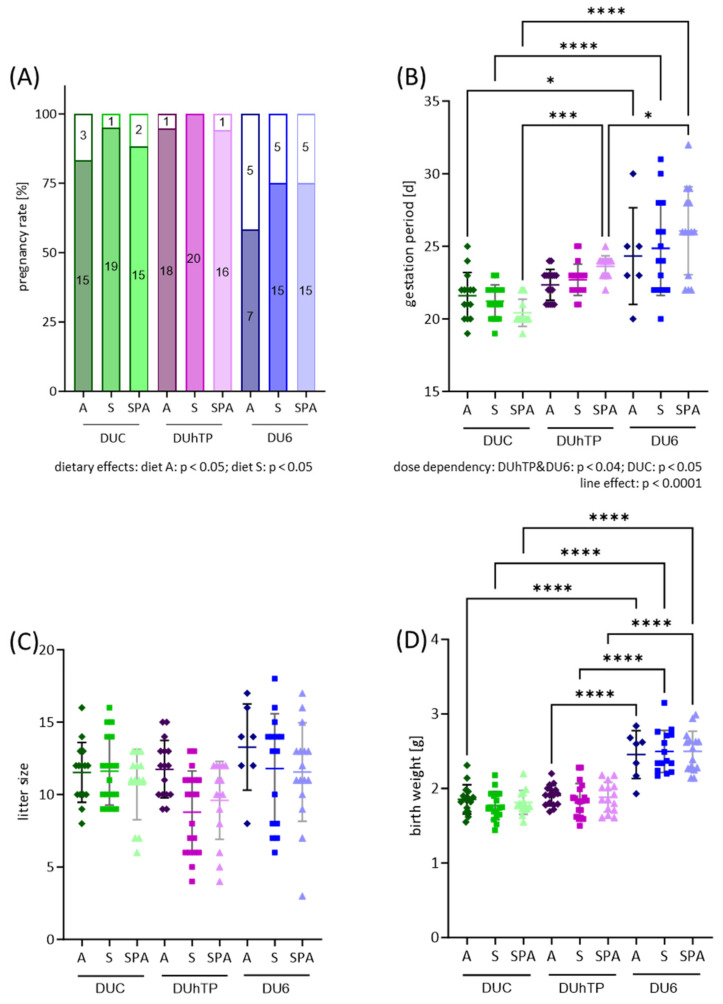
Effects of genetic background (line) and diet on reproductive performance in female mice. Successful mating was assumed by the presence of a vaginal plug and dated as DOP1 (first day of pregnancy). (**A**) Pregnancy was determined by the delivery of offspring within 35 days. The pregnancy rate is shown as a percentage bar chart, with integrated numbers of successful and unsuccessful pregnancies. (**B**) Duration of gestation in female DUC (n ≥ 14), DUhTP (n ≥ 16), and DU6 (n ≥ 6) mice from DOP 1 to birth. Gestation is shown in days as a scatter dot plot, each dot representing one pregnant female mouse. (**C**) The average litter size of DUC (n ≥ 13), DUhTP (n ≥ 15), and DU6 (n ≥ 7) mice are shown as a scatter dot plot, each dot representing the size of an individual litter. (**D**) Average offspring birth weights are shown in a scatter dot plot; each dot represents the average body weight of an animal per litter. Colors indicate mouse strain: green—DUC, purple—DUhTP, blue—DU6. The shade of color indicates the chow group: dark—diet A, medium—diet S, and light—diet SPA. Statistical analysis for (**A**) pregnancy rate was performed using a two-tailed Chi-Square test and a two-way ANOVA for (**B**–**D**). Significant differences are indicated as follows: * *p* < 0.05, *** *p* < 0.001, **** *p* < 0.0001. For abbreviations, see Figure 1.

Within the chow groups, two mating rounds were performed to obtain the “generation 2 animals” (see Figure 1). In response to the different IF-containing diets, no significant changes in pregnancy rate, gestation length, litter size, and birth weight were observed within each mouse line after the second mating (Figure 2A–D). However, a negative dose dependency was found in the DUhTP and DU6 mice (Figure 2B; *p* < 0.05), showing a shorter gestation period at higher dietary IF levels. In the DUC mice, the gestation period increased with increasing IF concentration in the diet (*p* < 0.05). 

Between the lines, significant differences were observed in terms of pregnancy rate (Figure 2A), length of gestation (Figure 2B), and birth weight (Figure 2D). Diet A and diet S, in particular, had a significant impact on the pregnancy rate (Figure 2A; both *p* < 0.05). While a 100% pregnancy rate was observed in DUhTP_S_ mice in this generation studied, the pregnancy rate in the DU6 animals was a maximum of 75% and as low as 58% when diet A was administered. Thus, the pregnancy rate of the DU6 mice was lower than that of the DUhTP (*p* < 0.05) and DUC mice (*p* < 0.055).

In contrast, the DUC animals had the shortest gestation period of around 21 days, followed by the DUhTP mice, with a duration of 23 days, and the DU6 mice, with 25 days (*p* < 0.0005, independent of the diet). DU6 displayed a longer gestation duration than DUC mice (*p* < 0.05). Furthermore, gestation durations differed significantly between SPA-fed animals of all lines (*p* < 0.05). 

While the litter size did not differ between the lines, the birth weights of the DUhTP and DUC animals were significantly lower than those of the DU6 mice independent of the diet (*p* < 0.0001; Figure 2D). 

### 3.3. Body Weight and IF Concentrations in Plasma from Juvenile Female Mice 

In the second generation, eight 21-day-old females per group and line were analyzed (“juvenile females”). As expected, the DU6 females (diet A: 26.6 ± 3.3 g, S: 27.6 ± 1.8 g, SPA: 25.2 ± 5.6 g) were significantly heavier than the DUC (diet A: 13.9 ± 0.8 g, S: 13.9 ± 0.7 g, SPA: 13.2 ± 1.4 g) or DUhTP females (diet A: 12.4 ± 1.3 g, S: 9.9 ± 1.2 g, SPA: 11.8 ± 1.2 g; *p* < 0.0001). When diet S was administered, the body weights of the DUhTP mice were also significantly lower than those of the DUC mice (*p* < 0.05).

Analysis of plasma IF concentrations revealed no relevant IF concentrations in most mice fed a phytoestrogen-free diet (SPA). Although IF concentrations were occasionally found in the plasma of the juvenile animals, these could be identified as outliers using the ROUT method (Q = 0.2%; DUC_SPA_: n = 2 outliers, DUhTP_SPA_: n = 2 outlier). The plasma of the DU6 mice fed diets A and S exhibited significantly higher IF levels (total IFs, total daidzein equivalents, free daidzein, total genistein equivalents, and free genistein equivalents) than DUhTP mice or DUC mice (*p* < 0.0001; Table 2). In particular, DUhTP_A_ had 30% and DUC_A_ had 57% lower total plasma IFs than DU6_A_ mice, and DUhTP_S_ had 47% and DUC_S_ had 55% lower plasma IFs than DU6_S_ mice, respectively. In both DUhTP and DU6 mice, significantly higher total IF concentrations were detected in the plasma of diet A-fed mice than in those fed diet S (+78%, *p* < 0.001 and +35%, *p* < 0.005). Similar significant differences between DUhTP_A_ and DUhTP_S_ or DU6_A_ and DU6_S_ were observed for plasma concentrations of free daidzein aglycone (+193%, *p* < 0.008 and +150%, *p* < 0.0001), total daidzein equivalents (+127%, *p* = 0.002 and +105%, *p* < 0.0001), free genistein aglycone (+259%, *p* < 0.005 and +141%, *p* < 0.0001), and total genistein (+140%, *p* < 0.001 and +116%, *p* < 0.0001), while in DUC mice no significant differences were detectable. In contrast, equol plasma concentrations (sum of equol, equol-7-glucuronide, and equol-4′-sulfate) did not differ significantly between diet A and S in DUC and DUhTP mice. In DU6 mice, however, the administration of diet S resulted in increased equol plasma concentrations compared to diet A (+48%, not significant) and diet S-fed DUhTP and DUC mice. Generally, equol concentrations were very similar between DUhTP_A_ and DUhTP_S_ and DUC_A_ and DUC_S_ mice, respectively.

### 3.4. Effects of Genetic Background and Diet on Reproductive Development in Juvenile Female Mice

**Figure 3:**
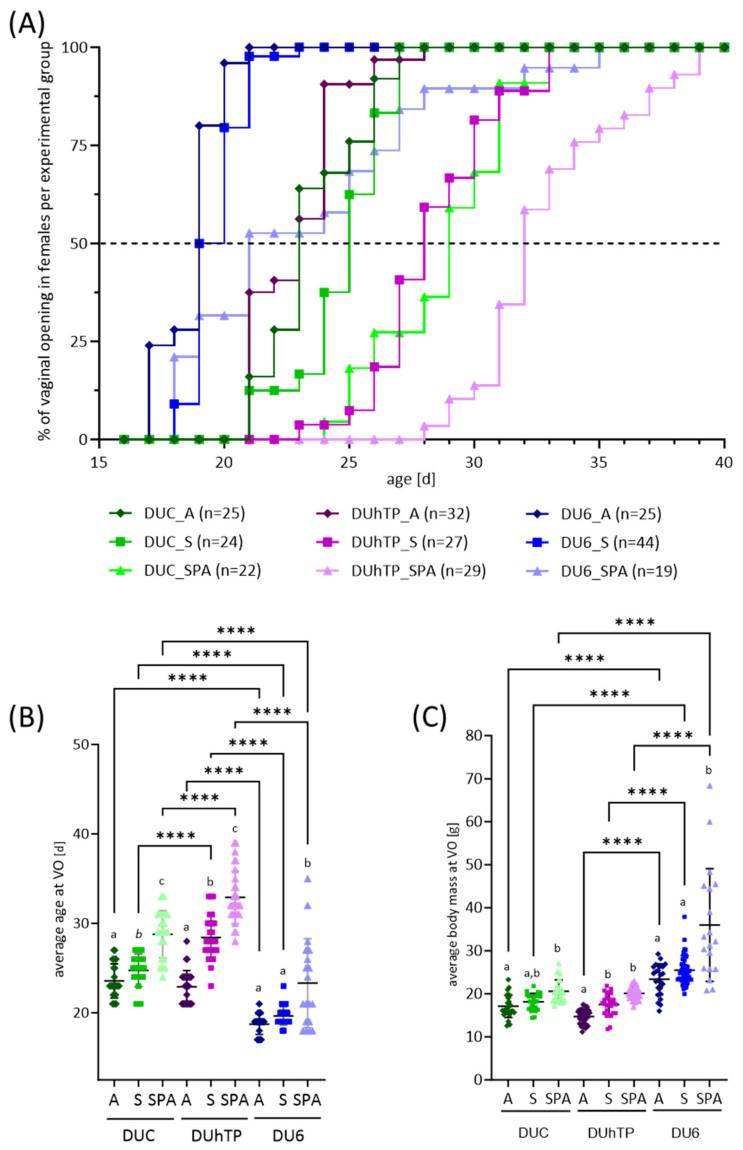
Effects of genetic background and diet on reproductive development in juvenile female mice DUC (green), DUhTP (purple), and DU6 mice (blue) fed with diet A (dark-colored), S (medium-colored), and SPA (light-colored). The onset of puberty in young female mice was identified by determining the age [d] at vaginal opening. (**A**) Puberty onsets over time in the different lines and feeding groups were plotted as a staircase graph. The dashed line indicates the day when 50% of the mice in each group (median) had an open vagina. (**B**) Average age and (**C**) average body mass on the day of vaginal opening are demonstrated as dot plots. The number of animals examined (n) is given in Figure A. Statistical analysis was performed using two-way ANOVA. Significant differences between the mouse lines are indicated as **** *p* < 0.0001. Diet-associated differences within the lines are marked with different letters, italicized in (**B**) at *p* = 0.07. For abbreviations, see Figure 1.

The onset of puberty was assessed by determining the age at vaginal opening. Between the genetic groups, we could identify differences in the onset of puberty, with puberty starting earlier in the heavy DU6 mice than in the other lines (genetic overall effect, *p* < 0.0001; Figure 3A). Furthermore, the diet had an IF dose-dependent effect on puberty onset (Figure 3A,B; *p* < 0.0001), showing earlier VO under diet A (DUC: 24 ± 2 days, DUhTP: 23 ± 2 days, DU6: 19 ± 1 days), followed by diet S (DUC: 25 ± 2 days, DUhTP: 28 ± 2 days, DU6: 20 ± 1 days), and diet SPA (DUC: 29 ± 3 days, DUhTP: 33 ± 3 days, DU6: 23 ± 5 days) (Figure 3B). In DUhTP (*p* < 0.05) and DUC mice (*p* ≤ 0.07), the timing of VO under diet S was significantly delayed compared to diet A but was accelerated compared to diet SPA (Figure 3B). When half of the females in each group had an open vagina (median, see dotted line in Figure 3A), a delay of two (diet S, *p* = 0.07) and four days (diet SPA, *p* < 0.0001) was observed in DUC mice and of five (S, *p* < 0.0001) and four days (SPA, *p* < 0.0001) in the DUhTP mice compared to the DUC_A_ and DUhTP_A_ mice, respectively (Figure 3A,B). The DU6 mice fed diet A and diet S were almost the same age on the day of VO, while the DU6 females fed the SPA diet were, on average, three days older (*p* < 0.05). Significant differences between the DUC and DUhTP mice were not observed. However, the body weight of DUhTP_A_ was 2.5 g lower than in DUC_A_ at VO (*p* < 0.1).

**Figure 4:**
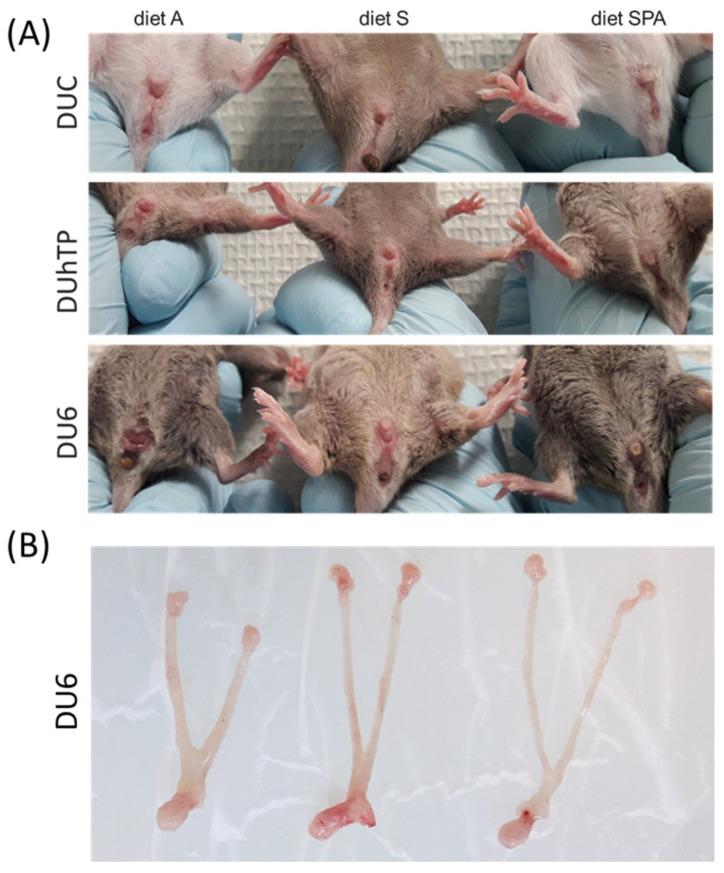
Effects of genetic background and diet on reproductive development in juvenile 21-day-old DUC (first row), DUhTP (second row), and DU6 females (third row) that received diet (**A**) (left), S (middle), or SPA (right). The photographs show (**A**) the outer genitals and (**B**) the ovary, uterine horns, and uteri of diet A (left), S (middle), or SPA (right)-fed DU6 mice. For abbreviations, see Figure 1.

Since body weight is associated with the onset of puberty [60], we also compared the body weights of the mice of each group at VO. A general genetic effect was detectable between the mouse lines regarding body mass at the age of VO (*p* < 0.0001, Figure 3C), as already seen at 21 days of life. However, significant feeding group-related changes in body weights at VO were observed, similar to the ages at VO (Figure 3B). That means, for all lines, the animals with a later onset of puberty due to their diet and, therefore, with higher age, also had a higher body weight. In particular, mice fed an SPA diet were characterized by increased body mass at VO. They were significantly older than mice of the same line fed with diet A or diet S (*p* < 0.0001, Figure 3B) and heavier than diet A-fed mice of the same line (*p* < 0.02, Figure 3C). DU6_SPA_ mice showed an even higher body weight on the day of VO than DU6_S_ mice. However, DU6_A_ and DU6_S_ mice had a similar body mass and age at VO. At 21 days of age, almost all DU6_A_ and DU6_S_ displayed VO (Figure 3C and Figure 4A), with DU6_A_ mice exhibiting shortened and thickened uterine horns and uteri (Figure 4B).

### 3.5. Effects of Genetic Background and Diet on Body Weight, Reproductive Organ Weights, and Feed Intake in Adult Female Mice 

Adult females were mated at around 69 days of age, and successful mating was evaluated by the presence of a vaginal plug. At DOP 4, three days after observing the plug, mice were analyzed. DOP 4 was chosen to minimize the effects of individual endogenous estrogen fluctuations during the menstrual cycle [61]. At this time, mice were, on average, 74 days old (DUC: 73 days, DUhTP: 81 days, DU6: 69 days), demonstrating a duration of around five (DUC, DUhTP) and six days (DU6) between mating and DOP4. 

In terms of body mass, adult DU6 mice (88.6 ± 7.8 g), like their juvenile siblings, were significantly heavier than DUC (33.2 ± 3.2 g, *p* < 0.0001) and DUhTP mice (30.1 ± 2.4 g, *p* < 0.0001; Table 3). For their part, DUC mice were heavier than DUhTP mice (*p* < 0.05). More precisely, DUC_S_ females had a significantly higher body weight than DUhTP_S_ females (*p* < 0.05), as already observed in juvenile sisters. Interestingly, the average body weight of the DUhTP_S_ mice was 10% lower than that of the DUhTP_A_ mice. However, this difference was not statistically significant.

**Figure 5:**
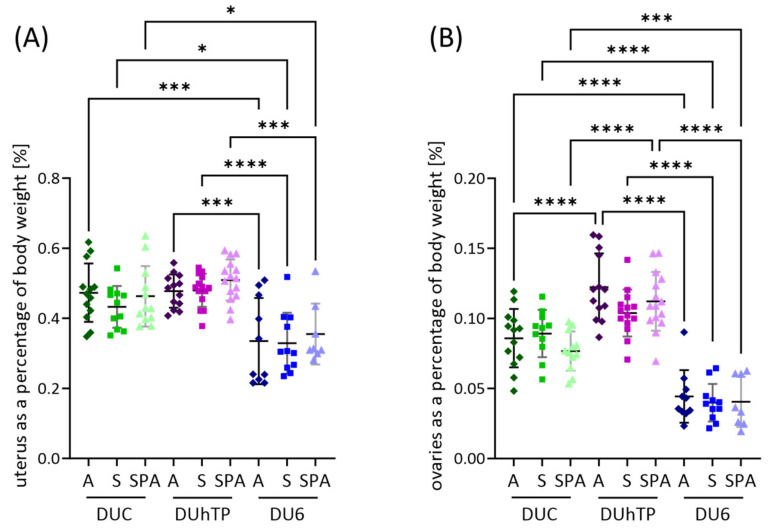
Effects of genetic background and diet on (**A**) relative uteri weights and (**B**) relative ovarian weights in adult female mice DUC (green), DUhTP (purple), and DU6 mice (blue) fed with diet A (dark-colored), S (medium-colored), and SPA (light-colored). The number of animals examined (n) is given in Table 3. Statistical analysis was performed using two-way ANOVA. Significant differences between the mouse lines are indicated as * *p* < 0.05, *** *p* < 0.001, and **** *p* < 0.0001. For abbreviations, see Figure 1.

Uterine weights were also higher in DU6 mice than in DUC (*p* < 0.0001) or DUhTP mice (*p* < 0.0001), while ovarian weights showed no differences (Table 3). However, when organ weights were considered in relation to body weight (Figure 5A), DU6 mice had lower uterine (Figure 5A) and ovarian (Figure 5B) weights as a proportion of body weight than DUC (*p* < 0.05) and DUhTP animals (*p* ≤ 0.0005). In addition, when fed diet A or SPA, the smaller DUhTP mice had higher relative ovarian weights than the DUC and DU6 animals (*p* < 0.0001).

The daily feed intake was less in the DUhTP mice compared to the DUC or DU6 mice in all diet groups (*p* < 0.005; Table 4). The DUhTP_SPA_ mice consumed more than the DUhTP_A_ and DUhTP_S_ mice (*p* < 0.05). In contrast, the DU6 mice consumed twice as much feed as the DUC and DUhTP mice (*p* < 0.0001), and the DU6_A_ mice ingested more than the DU6_S_ mice (*p* < 0.05). Since the DU6 mice have an increased weight and size due to selection, a higher food intake was not surprising. Nevertheless, in relative terms, the DU6 mice consumed only about 14.9% of their body weight in food per day, less than the DUhTP (18.2%) and DUC mice (16.9%). 

To be more precise, daily calorie intake in kcal per gram body weight was calculated based on the weekly measured food intake, chow calories, and animal weight. We observed a significantly lower calorie intake per gram body weight of the DU6 mice compared to the DUhTP mice (*p* < 0.01, *p* < 0.0001, *p* < 0.0001) and the DUC mice (*p* < 0.05, *p* < 0.0005, *p* < 0.0001) when fed diet A, S, or SPA (Table 4). 

### 3.6. IF Concentrations in Plasma from Adult Female Mice 

The plasma IF concentrations were also determined in the older animals (Table 5). In general, no relevant IF concentrations were detected in the plasma samples of mice fed with phytoestrogen-free chow (SPA). The ROUT method was applied to identify outliers (Q = 0.2%; DUC_SPA_: n = 1, DUhTP_SPA_: n = 4, DU6_SPA_: n = 2 outliers). Almost no differences in total IF and IF equivalent concentrations were found between the DUC and DUhTP mice fed with diet A or diet S. Merely, total daidzein equivalents were only increased in the plasma of the DUhTP_A_ mice compared to the DUC_A_ (*p* < 0.05) and DUhTP_S_ mice (*p* < 0.001). Generally, IF concentrations were very similar between the DUC_A_ and DUC_S_ mice, while concentrations differed between the DUhTP_A_ and DUhTP_S_ and DU6_A_ and DU6_S_ mice by a factor of around two. Only the sum of equol equivalents within all lines in feed group S is about 45% less concentrated than in feed group A.

However, in the DU6 mice, higher concentrations of total IFs, total daidzein and genistein equivalents, daidzein and genistein, and the sum of equol, equol-7-G, and equol-4′-S were detected in the plasma of mice fed a higher IF content diet (diet A) than in those fed diet S. Furthermore, the plasma concentrations of total IFs, daidzein, genistein, and genistein equivalents in the DU6_A_ mice were significantly higher than those in the DUhTP_A_ mice and DUC_A_ mice. Levels of total daidzein equivalents in the DU6_A_ mice differed from those in the DUC_A_ mice and the sum of equol, equol-7-G, and equol-4′-S from those in the DUhTP_A_ mice. In contrast, when the DU6 mice were fed diet S, they were characterized by higher plasma concentrations of free daidzein and genistein than the DUhTP_S_ mice. Apart from the mice fed with phytoestrogen-free chow (SPA), the lowest concentrations of free daidzein and genistein were detected in the plasma of the DUhTP_S_ mice.

### 3.7. Effects of Genetics and Diet on the Expression of Estrogen Receptors in the Ovaries

**Figure 6:**
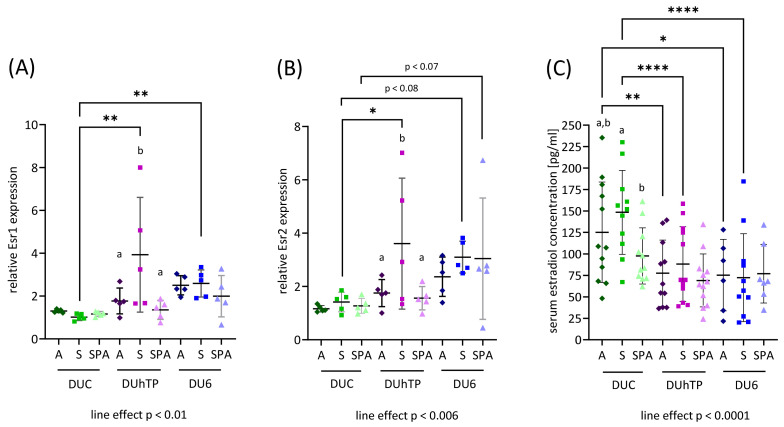
Relative expression of (**A**) estrogen receptor 1 (Esr) and (**B**) estrogen receptor 2 mRNA (Esr2) in ovaries of adult DUC (green), DUhTP (purple), and DU6 (blue) females determined by RT-qPCR (n = 5 each). (**C**) Plasma estradiol concentration was measured by ELISA (n = 5 each). The shade of color indicates the chow group: dark—diet A, medium—diet S, and light—diet SPA. Data are visualized as scatter plots. Statistical analysis was performed using two-way ANOVA. The line effect was determined using one-way ANOVA. Significant differences are indicated as follows: * *p* < 0.05, ** *p* < 0.01, and **** *p* < 0.0001. For abbreviations, see Figure 1.

To further study the differences observed in reproductive development and ovarian weight between strains and chow groups, the relative expression of Esr1 and Esr2 in ovaries was determined by RT-qPCR. Both Esr1 and Esr2 expressions generally differed line-specifically (Figure 6A,B), showing the lowest expressions in the ovaries of DUC females (*p* < 0.01, line effect). The highest Esr1 and Esr2 expressions were observed in DUhTP_S_ ovaries, significantly different from DUhTP females fed diet A or SPA (*p* < 0.05). Apart from that, the DUC_S_ mice showed significantly lower Esr1 and Esr2 expression levels than the DUhTP_S_ (*p* < 0.0005 and *p* < 0.05) and DU6_S_ mice (*p* < 0.05 and *p* = 0.079), respectively. There was also a tendentially higher expression of Esr2 in the DU6_SPA_ compared with the DUC_SPA_ mice (Figure 6B; *p* = 0.062). Correlation analysis revealed a significant moderate impact of IF concentration on Esr2 expression in the DU6 mice (*p* < 0.05; Table S3).

We further measured plasma 17β-estradiol levels, which were highest in blood samples of the DUC mice (Figure 6C; *p* < 0.0001, line effect). In comparison to DUC mice fed diets A and S, the DUhTP_A_ (*p* < 0.005) and DUhTP_S_ mice (*p* < 0.0001), as well as the DU6_A_ (*p* < 0.005) and DU6_S_ mice (*p* < 0.0001), had significantly lower blood estradiol levels. Furthermore, the DUC_S_ mice had higher estradiol levels than the DUC_SPA_ mice (*p* < 0.001). Interestingly, we could not detect estradiol in some DU6 and DUhTP mice plasma samples. In nine DU6 and six DUhTP mice, the measured values were below the measurement limit of 20 pg/mL, whereby there was apparently a negative frequency of low values depending on the IF content. In the groups receiving diet A, there were more frequent samples with measurements below the detection limit (DU6: 5, DUhTP: 3) than with diet S (DU6: 3, DUhTP: 2) or diet SPA (DU6: 1, DUhTP: 1).

## 4. Discussion

This study describes the effects and interactions of diet and genotype on reproductive development in mouse models. Two different commercially available chows (Altromin (diet A) and Ssniff (diet S)) and a phytoestrogen-free feed (diet SPA; Ssniff) with similar macronutrient profiles were used in mouse models representing different metabolic phenotypes (DUC—control, DUhTP—high metabolic activity, and DU6—obese). Our results indicate that the diets characterized by different IF contents affect reproductive development in mice and that this effect differs due to the metabolic phenotype of the respective mouse line.

### 4.1. Commercially Available Chow is Isoflavone-Rich and Causes Therapeutic Plasma IFs

Analysis of the standard chows revealed a relatively high total IF aglycone content in diets A and S, with the concentration in feed A being fifty percent higher than in feed S, consistent with a previous study using diets from the same manufacturers [62]. Furthermore, these concentrations are in accordance with the manufacturer’s ingredient lists. In diet A, soy is the main source, which contains large amounts of isoflavones, followed by wheat and corn. In contrast to that, feed S consists mainly of wheat, containing hardly any phytoestrogens [63], followed by soy products and corn. While diet S could be considered an intermediate-IF chow within our study, it must be noted that an overall IF aglycone content of 0.4 mg/g can still be regarded as relatively high. So, diets A and S should rather be perceived as very high and high IF-containing diets. 

This high IF content resulted in a daily dose of approximately 55–93 mg/kg body weight, leading to mean total plasma IF concentrations of 1.9–6.7 µmol/L and genistein levels of 0.5–2.6 µmol/L in our juvenile and adult mice. For comparison with other studies, the dosage of orally administered genistein in a concentration between 0.4 and 100 mg/kg body weight resulted in serum IF levels of 0.8–5.8 µg equivalents h/mL or 2–3 µmol/L [25,29,64]. This indicates that biologically active plasma IF concentration can be achieved with commercially available standard chows. The results align with previous studies that found high steady-state serum IF concentrations of 2338 ± 531 ng/mL in mice, even exceeding the animal endogenous estrogen levels after feeding them commercially available rodent chow [65]. 

Remarkably, total IF concentrations in plasma were higher in adult DU6 animals compared to the other two lines. This was significant for the fed diet A, and, even if this was not significant for the fed diet S, the same tendency was observable. These findings may be explained by the higher feed intake of the DU6 animals, which was more than twice as high. However, in terms related to body weight, the IF dose was comparable between the lines. To compare the IF plasma concentrations across the lines, a direct correlation between calculated IF dose and plasma IF concentrations might not be suitable since the food intake was recorded just weekly. Additionally, the distribution volume, an essential parameter in biokinetics influencing plasma levels, is not comparable between the lines, especially when comparing DU6 mice (relative high fat mass) with the other lines. 

Regarding the juvenile mice, a similar observation was made with the highest total plasma IF concentrations in DU6 animals. In juvenile animals, no food intake was recorded. Still, it might be speculated that these animals started earlier in life with fed diet consumption due to the overall observed accelerated development of DU6 mice compared to the other two lines. In this context, it might be further speculated that this led to higher IF plasma levels in the early life of DU6 mice because of the longer repeated intake of IF, reaching the steady state concentrations of IF earlier. However, this study aimed not to conduct a biokinetic investigation, and the observations regarding plasma IF concentrations would have to be examined in further studies. 

Equol-7-G and equol-4′-S could be detected in almost all juveniles, except one DUC_A_ (no equol-7-G) and one DU6_A_ female (no equol-4′-S), and all adult animals fed with diet A and S. This indicates that the microbiota of the mice even at a young age were capable of metabolizing daidzein to equol. This is in line with another study detecting equol-7-G and equol-4′-S in the serum of male and female BALB/c mice (age around 8 weeks) after intake of a phytoestrogen-free diet enriched with an IF-containing soy extract [66]. Equol exhibited a higher estrogenic potential than daidzein. The individual rate of metabolism of daidzein to equol (different IF metabotypes) may lead to different estrogenic potencies of ingested IFs, as discussed in humans [6]. However, the specific role of equol and its phase-II-metabolites for the observed effects in our study cannot be evaluated since a complex profile of IF metabolites with different estrogenic potencies was detected in the plasma of mice but not locally in the tissues.

### 4.2. Dietary Effects on Reproductive Performance and Interactions with Genotype

Generation 1 mice were mated within their chow group, and the occurrence and duration of pregnancy, litter size, and individual birthweight were used as markers for fertility. Although all three lines originated from the same genetic background, strain-specific differences were detectable, mainly affecting the DU6 line. The DU6 mouse line, being almost three times heavier than the control DUC mice [51] and, to our knowledge, the heaviest mouse line worldwide, is characterized by a massive adipose phenotype [67]. Probably due to general obesity, the DU6 females had poorer pregnancy rates, longer gestation periods, and higher birth weights than the control line, as also described by Palma-Vera et al. [51], with comparable litter sizes. Similarly, diet-induced obesity in female mice increases infertility and offspring birth weight with largely unchanged litter size [68,69,70,71]. Interestingly, the DUhTP_SPA_ mice also demonstrated an enhanced gestation duration compared to control mice, but not as long as in the DU6_SPA_ mice. Since the DUhTP mice also exhibit a pronounced fat phenotype, although not as pronounced as in the DU6_SPA_ mice, this could be related to the longer gestation period. 

A positive correlation between maternal pre-pregnancy BMI and infertility [72], high maternal BMI and pregnancy duration [73,74,75,76], as well as maternal pre-pregnancy BMI and increased birth weight [77,78], respectively, has also been reported in humans. 

A prolonged pregnancy of four to five days has been described for women with a BMI of 40 kg/m^2^ [73]. The pathomechanism of this effect is not yet understood. However, it is likely that an increased amount of hormonally active adipose tissue, causing changes in endocrine factors (e.g., estrogen, progesterone, oxytocin, prostaglandins, corticotropin-releasing hormone, relaxin), is involved in the delayed induction of labor [79]. Although the effect of maternal obesity on pregnancy duration is probably similar in humans and mice, comparisons are limited as humans usually experience singleton pregnancies and not multiple pregnancies as rodents do. The positive correlation between maternal pre-pregnancy BMI and increased birth weight [77,78] may be partly related to the prolonged gestation duration, but other factors such as calorie supply, hormonal differences, and metabolic/health status (e.g., diabetes) may also play an important role. 

Nutritional influences on reproductive development have been the subject of many studies. Studies to date have often focused on the IFs daidzein and genistein, as they are the most abundant IFs in soybeans [32,33,64,80]. The studies suggested that exposure to IFs leads to impaired fertility. Jefferson et al. demonstrated reduced fertility of CD-1 female mice, characterized by fewer pregnancies and fewer live pups, that were subcutaneously treated with genistein of 25 or 50 mg/kg body weight from day 2 to 5 [32,33]. Similar exposure doses of approximately 27–44 mg/kg/day of genistein in aglycone equivalents, but in combination with other IFs, were used in our study, depending on the feed administered. While no significant differences in litter size or litter birth weight were observed, an effect of IFs, usually even dose-dependent, on the reproductive performance of the different mouse lines based on their metabolic habitus can be assumed. For example, the DU6 animals had a pregnancy rate of just over 50% when fed diet A, compared to 75% in animals that received a diet without IFs. DUC mice were characterized by an increasing gestation period with increasing IF concentrations. In contrast, DUhTP and DU6 mice have a longer gestation period, decreasing with increasing IF concentrations. 

Another study determined fertility in the outbred mice CD-1 after postnatal treatment with IFs [80]. For this, mice were fed an estrogen-free diet. The offspring were injected subcutaneously with 2 mg daidzein and 5 mg genistein per kg body weight within the first 10 and 21 days after birth, respectively, to represent the administration of soy-based infant formula during the early postnatal period and the maximum period during which humans should consume infant formula. The authors found slightly reduced pregnancy rates in generation 1 mice similar to DU6 but not DUC or DUhTP mice. Furthermore, no differences in gestation length, litter weight, or pup number were found in this study [80]. Of note, the mice were treated subcutaneously with 2 mg daidzein and 5 mg genistein per kg body weight for only 10–21 days postnatal, a much shorter exposure duration and lower concentration than in our study, in which 22–42 mg/kg/day daidzein and 27–44 mg/kg/day genistein were administered in the diet for 70 days, respectively.

Additionally, the method of exposure (subcutaneously vs. orally by diet) was different, which affected biokinetics. In C57BL/6 mice that received a genistein-soy formula emulsion at a concentration of 5, 20, 50, and even 100 mg/kg body weight during the first five days after birth, pregnancy rates and the number of live births were also not altered [64]. Although the results of these studies vary, it seems clear that the effects of IFs on reproductive performance depend on dosage form, concentration, and the mouse strain used (i.e., genotype).

Considering the limitations that humans and mice metabolize IFs differently [66], and that mice can only be used as models for humans up to a certain level, our study results may nevertheless provide some guidance for further human research. For example, in this study, we were able to show that, in obese female mice, an IF-rich diet leads to a further deterioration of the pregnancy rate. In previous studies that investigated the influence of dietary PE on female reproductive health, the phenotypic constitution of the subjects often played no or a subordinate role. In addition to other influencing factors, such as dose, duration of exposure, and age, the physical habitus of the test subjects or the mouse models used could also affect the different research outcomes. To our knowledge, our study is the first to address a potential mutual influence of physical/genetic habitus and dietary PE intake on reproductive development and fertility in females. 

### 4.3. IF-Containing Diets Accelerate Reproductive Development

There are multiple indicators for sexual maturation and development in rodent models. In females, vaginal opening (VO), an extensive apoptotic event occurring due to rapidly increasing estradiol secretion, is generally used to determine the onset of puberty and first ovulation, followed by the start of estrous cycling [81,82,83]. The vaginal opening can be determined by non-invasive visual examination of the vulva and usually occurs at ~26 days of age [48,84]. Other sources describe a broader range for the event from ~23 to 42 days for CD-1 [33,84], C57BL/6, or BALB/c mice [85,86]. It has previously been discussed that body weight, especially fat mass weight, may influence the age of puberty onset [39]. The minimum body weight/fat mass required to enter puberty is often called ”critical body weight” or ”critical fat mass”. It is thought to be around 20% fat mass of total body weight [41]. Furthermore, it has been shown that diet alterations, e.g., increased fat or calorie content, can accelerate puberty onset [82] and that IFs can delay or, more often, accelerate VO depending on doses, type, and duration of intake. Administration of 2 mg/kg genistein subcutaneously on days 1–6 and 40 mg/kg genistein orally on days 7–21 in rats, which corresponds to the daily dose consumed by our mice on diet A (39–44 mg/kg), resulted in an earlier onset of VO than administration of 0.2 mg/kg genistein subcutaneously and 4 mg/kg orally [29]. Orally administrated genistein (50 mg/kg body weight) during the first eight days postnatally accelerated VO compared to a bolus without the IF [30]. Subcutaneously applied genistein (0.5 mg/kg, 5 mg/kg, and 50 mg/kg BW) on days 1–5 resulted in no significant alterations in VO onset in CD-1 mice [33]. Four daily subcutaneous injections of 10 mg/kg/day of genistein from day 15 caused an earlier VO by 3 days in CD-1 mice [84]. 

We observed an average VO at 29, 33, and 23 days in DUC, DUhTP, and DU6 mice, respectively, when fed diet SPA, and found that different IF-containing diets accelerated VO in all three strains. A dosage-dependent IF effect on VO was observed in DUhTP and DUC mice. Mice fed a very IF-rich diet (A) were the youngest on the day of VO, followed by those fed an IF-rich diet (S), and those given the PE-free diet (SPA).

Interestingly, although both lines, DUC and DUhTP, are descended from the same original mouse line, paternal selection of DUhTP mice caused delayed female sexual development by four days, which was apparent when fed with diet SPA or S. Over time, the paternal selection on high treadmill performance has resulted in smaller and significantly lighter DUhTP mice than the unselected controls [51]. Thus, it could be related to reaching the critical body weight later because there is a significant difference in the age of VO onset between the DUhTP and DUC mice but not in the body weight at VO onset.

This difference in the onset of puberty no longer existed between the DUhTP and DUC mice when diet A was administered. Accordingly, the administration of diet A with a higher IF content had a higher impact on the onset of puberty in the DUhTP mice than in the DUC mice, since the body weights of the DUhTP_A_ mice were lower than those of the DUC_A_ mice at VO, although this difference was not statistically significant. Previous studies with diet A have shown that DUhTP mice differ not only from controls in their low body weight but also show a significantly increased mass of brown and beige adipose tissue [87]. These tissues are considered to be highly metabolically active. Both tissues have increased mitochondrial mass and express uncoupling protein 1 (UCP1) [88], which uncouples ATP synthesis from respiration, resulting in the dissipation of energy in the form of heat [89]. This trait leads to an increased surface temperature [54] and potentially to a higher basal metabolic rate. These phenotypic and genetic traits may make DUhTP female mice more sensitive to exogenous/dietary PE than DUC mice. Since DUhTP mice were paternally selected based on high running performance, they could be considered athletic. In humans, it is known that very athletic girls tend to have a delayed onset of puberty [39,40,41,42,43,44,45], so we can draw parallels with our DUhTP mice and their later onset of puberty as long as a low dietary IF concentration is provided. Although athletic girls are characterized by a reduced body fat percentage compared to our mice, our data suggest that administering high IF doses can accelerate the onset of puberty in this “athletic” type.

DU6 mice generally had a much earlier onset of puberty, which may be related to the obese phenotype of DU6 mice. That suggests that they (1) reach “critical body weight” relatively early and (2) accumulate large amounts of adipose tissue, which functions as an endocrine organ and, therefore, helps to regulate the onset of puberty [90,91]. IFs in the diet caused an earlier onset of puberty by three days, with no difference between very high IF (A) and high IF (S) exposure. It is possible that both earlier attainment of critical body weight and increased fat accumulation could superimpose the influence of a dose-dependent effect of IFs on puberty onset in DU6 mice. 

Nonetheless, consistent with the “critical body weight” theory, the obesity of DU6 mice could be responsible for the fact that the mice generally reach puberty earlier than the other lines, regardless of the food group. Similar observations concerning higher body mass and earlier VO were made in a recent study by Gomes et al. in which genetically obese BPH/5 female mice displayed VO at 21 days of age compared to puberty onset at 29 days in C57BL/6 controls [92]. Additionally, Bohlen et al. showed that prepubertal obese C57BL/6 female mice, induced by overnutrition, displayed a significantly earlier VO (28 days) at a weight of approximately 15 g than normal-weight littermates (33 days, 13 g) [49]. Thus, both genetically obese DU6 mice and overnutrition-induced obese C57BL/6 females opened vaginally five days earlier than DUC and normal-weight C57BL/6 females, respectively. However, the C57BL/6 mice differed in weight by only 15% at these time points. In contrast, the Dummerstorf lines differed by nearly 40%, reflecting the massive fat accumulation due to selection progress. This accumulation could also substantiate the second theory that the adipose tissue, especially the considerable amount found in DU6 mice, acts as an endocrine organ secreting adipokines, especially kisspeptins and leptin [93]. Previous studies have shown that hypothalamic kisspeptin signaling is crucial for sexual maturation and reproduction. Leptin and kisspeptin are, therefore, often referred to as “gatekeepers” of the hypothalamic–pituitary–gonadal axis [94,95,96,97,98]. Leptin is attributed a similar significance by stimulating kisspeptin neurons to interact with gonadotropin-releasing hormone (GnRH) neurons or in the pituitary, directly stimulating the production of GnRH receptors [99]. A separate study is dedicated to the in-depth investigation of the connections between adipose tissues, serum leptin concentrations, and kisspeptin expression in the mouse models described here. 

Our results suggest that an IF-rich diet accelerates female pubertal development in all lines studied. The extent of the influence of IFs on sexual maturation appears to depend strongly on the genetic group and phenotype. It is noteworthy that the differences in the onset of puberty between obese DU6 mice and mice from the other groups are substantial (from six to ten days) in the IF-free diet (SPA) but minimal (four days) in diets with a very high IF content (diet A). The reason for this is that an IF-rich diet shortens the onset of puberty in overweight mice by only four days, in mice with high metabolic activity by ten days, and in control mice by five days. Interestingly, there is only one day between the onset of puberty in DU6 mice, two days in DUC mice, and five days in DUhTP mice between diets with high (diet S) and very high IF content (diet A). This could be explained by the total serum IF content, which is twice as high in DUhTP mice fed diet A compared to diet S, while the difference in serum IF content in DUC and DU6 mice is a factor of about 1.3. This greater increase in plasma IF concentration between diets S and A in DUhTP mice than in DUC and DU6 mice may explain the faster puberty onset by five days in DUhTP mice and, therefore, no difference in the onset of puberty between DUhTP_A_ and DUC_A_ mice. The reasons for the greater increase in juvenile DUhTP_A_ mice are unknown. They would need to be investigated in subsequent studies. However, a higher food intake in the DUhTP_A_ mice is unlikely, as we found no differences in calorie intake in the older siblings, and both lines reached puberty at the same time, meaning that they consumed the high IF food for a comparable time after weaning.

### 4.4. Reproductive Organ Weights, Estrogen Receptor Expression, and Serum Estradiol

Besides the non-invasive determination of age at VO to define puberty onset, invasive methods can also be used to test endocrine activity and sexual development. These methods include measurement of serum sex steroid hormones (e.g., estradiol) to detect estrogenic changes in the endocrine system, sex organ weight, with higher weights of uteri and ovaries indicating estrogenic effects due to higher cell proliferation rates, and estrogen receptor expression and activity, particularly in the ovaries, as increased expression of Esr1 and 2 indicates higher estrogenic activity [25]. It is known that IFs bind to estrogen receptors, whereby IFs have a higher binding affinity for Esr2 than for Esr1 [11,100,101,102], and Esr2 transcripts predominate over Esr1 transcripts in ovaries [103].

Therefore, uterine and ovarian weights, as well as ovarian Esr1 and Esr2 expression and plasma estradiol concentrations, were determined in adult female mice at DOP 4. 

In general, DU6 mice had higher uterine and ovarian weights than DUhTP and DUC mice, which can be explained by the overall larger size and higher body weight of DU6 mice. However, relative to the body weight, the DU6 uteri and ovaries had a lower weight than those of DUC or DUhTP mice because the ratio between organ and body weight is different in DU6 animals than in the other lines due to the massive peripheral fat accumulation. IF-mediated effects on uterus weight were not found in our strains. It was previously demonstrated in CD-1 mice that relatively high amounts of genistein administrated by subcutaneous injections forthree to five consecutive days lead to increased uterine wet weight with increased uterine epithelial cell height and number of glands [31]. These increases were not observed when CD-1 mice were subcutaneously exposed to the same concentration of daidzein [31]. In our study, the mice received a wide range of naturally occurring IFs due to oral ad libitum administration of diets characterized by simultaneously high concentrations of genistein and daidzein. Non-oral administrations, such as intravenous or subcutaneous injection, circumvent the “first-pass effect”, which can lead to a lesser extent of the metabolization of IFs to phase II conjugates. These metabolized IFs have an altered estrogenic potency. For example, IF glucuronides exhibit a lower estrogenic activity than their respective aglycones [104,105]. This may explain why no effects on uterine weight were observed in our study. 

Interestingly, the weight of the ovaries in DUhTP mice was significantly altered by the diet in line with their body weight, demonstrating the highest ovarian and body weights when fed with diet A. However, DUhTP mice generally were characterized by higher relative ovarian weights, followed by DUC and DU6 mice. 

Estrogen receptor expression, indicative of possible higher estrogenic activity [25], displayed higher expressions in DUhTP_S_ than in DUhTP_A_ mice. In contrast, very low Esr1 and Esr2 expression levels were detected in the ovaries of DUC females. The integration of estrogen receptor signaling is thought to regulate cell proliferation, differentiation, motility, and death, depending on the estrogen-target tissue [106]. Both estrogen receptors are structurally able to bind 17-β-estradiol and phytoestrogen metabolites. Estrogen receptor binding leads to dimerization and attachment of the receptor–ligand complex to the estrogen response elements (EREs) in the promotor region of target genes, influencing their expression [11,100,101,102]. Due to their competitive binding to estrogen receptors, the estrogenic and anti-estrogenic effects of IFs depend on the coexisting estrogen/estradiol concentration. 

In our study, we detected high plasma estradiol levels in all lines, with higher concentrations in DUC than in DUhTP or DU6 females. We attribute this observation to the steadily increasing estradiol concentrations during pregnancy found in humans [107], mice [108], and rats [109]. Interestingly, similar estradiol levels were observed in all lines when IF-free feed was administered. IF-containing feed, especially diet S, markedly increased estradiol levels only in the control mice DUC. Since IFs generally show rather an antagonistic effect at physiological estrogen/estradiol levels, as their binding to the estrogen receptors is less strong than that of endogenous estrogen [10,11,12], we can assume an antagonistic effect of IFs in these diet S-fed control mice. 

This differs in the DOP 4 DU6 and DUhTP animals, where low estradiol levels and even samples below the detection limit were measured, especially in the animals fed diet A. At low estrogen concentrations, as in DU6_A_ mice, IFs can act as ER agonists [11,12], having a major impact on body composition [13,14,15], metabolism [16,17,18,19,20,21,22,23,24], and fertility [25]. Although this combined impact of estrogen and IFs on the reproductive maturation and development of females was not explicitly examined in this study, phenotype-associated effects can be assumed since consuming the same diets to a comparable extent resulted in different plasma concentrations of IFs and estradiol, even in similarly sized animals such as the DUC and DUhTP mice.

With the study design conducted here, it is impossible to attribute the observed effects to distinct IFs, as our analyses focus specifically on the effects of IFs as components of standard diets. Although diets with similar macronutrient profiles were chosen, it cannot be excluded that other dietary components, as well as the source of IFs, also affect or at least influence the study parameters. Sex-specific differences in IFs should also be considered in future studies by investigating the effects of IFs on male reproductive development. 

## 5. Conclusions

Mouse models that differ phenotypically and metabolically but are based initially on the same polygenetic background display different reproductive responses to diets with varying levels of isoflavones. Here, we demonstrate that IF-containing standard diets result in significant pharmacologically relevant plasma isoflavone concentrations, which have minor genotype-specific effects on reproductive performance but significant ones on reproductive development. Compared to a diet without IFs, a dose-dependent response of IFs on gestation length can be found in all lines and an effect on the pregnancy rate in DU6 mice. In addition, we can demonstrate an acceleration of the onset of puberty in all lines. Compared to the control line, we show a stronger effect on the onset of puberty in mice with high metabolic activity (DUhTP), which is generally characterized by delayed puberty, and a weaker impact in obese DU6 mice, which already show accelerated puberty.

Based on the plasma IF concentrations in the mouse lines, we conclude that the influence of circulating IFs is stronger in juvenile DUhTP and DU6 females than in their adult states, whereas it is the opposite in DUC mice, where the adults are more strongly affected than the juveniles. Especially diet S-fed DUC adults demonstrated comparable serum concentrations to diet A-fed DUC mice, although diet S has a lower IF content. Remarkably, higher estradiol levels can also be observed in diet S-fed adult DUC females. For the present study, we employed standard chows comprising a composition of different IFs. Therefore, the experimental setup did not allow the observed effects to be mechanistically assigned to the (inter)actions of individual substances. However, we showed that dietary phytochemicals, such as IFs, and metabolic phenotypes influence sexual development together rather than separately. This would mean that, in studies with mice, but also other species, the response to exogenous factors such as diet could vary drastically, and, as a consequence, phenotypic, reproductive, or other outcomes could be individually biased, even if the subjects are descended from the same original line. Our data suggest that, depending on the metabolic phenotype, IF-containing diets may affect metabolic pathways influenced by hormones (growth, sexual development) to varying degrees. Since the present findings may also be relevant to other vertebrates, including humans, more consideration should be given to biologically active secondary plant metabolites in diets.

## Figures and Tables

**Table 1:** Mean isoflavone contents of the standard chows A, S, and SPA in mg per g diet and percentage composition of the feeds related to total isoflavone aglycone equivalents, as determined by HPLC-DAD. Statistical analysis was performed using one-way ANOVA. Significant differences (*p* < 0.05) concerning content or percentage between the diets were marked using lowercase or uppercase letters, respectively. Abbreviations: A—Altromin, S—Ssniff, SPA—Ssniff phytoestrogen-poor alternative, n.d.—not detected/below quantitation limit.

Analytes	Diet A (n = 4)		Diet S (n = 4)		Diet SPA (n = 3)	
	Content [mg/g Diet]	Content (%)	Content [mg/g Diet]	Content (%)	Content [mg/g Diet]	Content (%)
Sum of Daidzein in Aglycone Equivalents	0.267	45.1 ^A^	0.157	39.9 ^B^	n.d.	-
Sum of Genistein in Aglycone Equivalents	0.287	48.2	0.195	50.2	n.d.	-
Sum of Glycitein in Aglycone Equivalents	0.041	6.8	0.039	10.0	n.d.	-
Overall Isoflavone Aglycone Equivalents	0.594 ^a^	100.0	0.392 ^b^	100.0	n.d.	-

**Table 2:** Plasma isoflavone concentration in nmol/L of juvenile (21 days) female DUC, DUhTP, and DU6 mice that received diet A, S, or SPA. Statistical analysis was performed using two-way ANOVA. Significant differences between strains compared to the DUC and DUhTP line are marked with # and §. Significant differences between chow groups are marked with letters. For abbreviations, see Figure 1; additional abbreviations: n.d.—not detected/below quantitation limit; G—glucuronide; S—sulfate.

Juvenile Females	Line	DUC			DUhTP			DU6		
	Diet Count	A n = 8	S n = 8	SPA n = 6	A n = 8	S n = 8	SPA n = 6	A n = 8	S n = 8	SPA n = 8
Total Isoflavones	mean	2475.3 ^a^	1939.8 ^a^	4.8 ^b^	4086.1 ^#a^	2291.1 ^b^	1.5 ^c^	5822.8 ^#§a^	4300.6 ^#§b^	n.d. ^c^
	SD	1142.5	746.1	8.8	666.4	510.7	2.3	1573.2	1464.1	
Total Daidzein Equivalents	mean	952.3 ^a^	656.5 ^a^	2.4 ^b^	1689.3 ^a^	745.8 ^b^	1.5 ^c^	2542.9 ^#a^	1241.8 ^#§b^	n.d. ^c^
	SD	809.2	309.9	4.1	365.0	163.3	2.3	1219.0	291.8	
Daidzein (free Aglycone)	mean	183.0 ^a^	102.7 ^a^	n.d. ^b^	215.7 ^a^	73.7 ^b^	n.d. ^c^	574.1 ^#§a^	229.5 ^#§b^	n.d. ^c^
	SD	154.1	54.2		74.9	22.5		195.3	41.9	
Total Genistein Equivalents	mean	718.2 ^a^	488.5 ^a,b^	2.0 ^b^	1353.9 ^#a^	564.0 ^b^	n.d. ^c^	2550.5 ^#§a^	1183.1 ^#§b^	n.d. ^c^
	SD	560.7	243.3	4.9	546.0	148.3		787.0	507.1	
Genistein (free Aglycone)	mean	74.5 ^a^	40.8 ^a,b^	n.d. ^b^	102.0 ^a^	28.4 ^b^	n.d. ^b^	307.5 ^#§a^	127.8 ^#§b^	n.d. ^c^
	SD	56.2	23.5		65.0	10.4		83.3	74.3	
Sum of Equol, Equol-7-G and Equol-4′-S	mean	804.9 ^a^	794.9 ^a^	1.5 ^b^	1042.9 ^a^	981.3 ^a^	0.3 ^b^	1036.9 ^a^	2003.6 ^#§b^	n.d. ^c^
	SD	640.2	281.9	3.1	237.0	300.2	0.8	729.5	1179.6	

**Table 3:** Phenotypic data of adult female DUC, DUhTP, and DU6 mice that received diet A, S, or SPA. Body mass and tissue weights of ovaries and uterus as means in g at DOP 4. Statistical analysis was performed using two-way ANOVA. Significant differences between strains compared to DUC and DUhTP are marked with # and §. For abbreviations, see Figure 1.

Adult Females	Line	DUC			DUhTP			DU6		
	Diet Count	A n = 13	S n = 11	SPA n = 12	A n = 13	S n = 14	SPA n = 15	A n = 10	S n = 11	SPA n = 8
Body Mass	mean	33.40	33.88	32.47	31.77	28.85 ^#^	29.75	89.95 ^#§^	89.10 ^#§^	86.04 ^#§^
	SD	3.57	2.51	3.58	2.45	1.77	2.25	8.21	4.96	10.58
Uterus	mean	0.157	0.146	0.149	0.152	0.139	0.151	0.297 ^#§^	0.293 ^#§^	0.307 ^#§^
	SD	0.024	0.015	0.029	0.020	0.016	0.018	0.098	0.080	0.092
Ovaries	mean	0.029	0.030	0.025	0.039	0.030	0.033	0.039	0.035	0.035
	SD	0.008	0.007	0.005	0.010	0.005	0.007	0.013	0.012	0.017

**Table 4:** Nutritional data of adult DUC, DUhTP, and DU6 females that received diet A, S, or SPA. Feed intake was measured weekly between the experimental days 28 and 65. Based on these data, the average daily feed intake in g was calculated. Average daily calorie intake to body mass (BM; Table 3) was calculated using food data. Statistical analysis was performed using two-way ANOVA. Significant differences *p* ≤ 0.05 are indicated as follows: hashtags (#) mark significant differences compared to DUC mice, and paragraphs (^§^) mark significant differences compared to DUhTP mice. Significant differences between chow groups are indicated by different letters. For abbreviations, see Figure 1.

Adult Females	Line	DUC			DUhTP			DU6		
	Diet Count	A n = 10	S n = 10	SPA n = 10	A n = 10	S n = 10	SPA n = 10	A n = 8	S n = 10	SPA n = 10
Feed Intake [g/d]	mean	4.62	4.49	4.66	3.94 ^#a^	3.91 ^#a^	4.21 ^#b^	11.04 ^#§a^	9.98 ^#§b^	10.50 ^#§a,b^
	SD	0.36	0.23	0.30	0.22	0.22	0.17	0.63	0.94	1.00
Calorie Intake [kcal/g BM/d]	mean	0.57 ^a^	0.57 ^a^	0.62 ^b^	0.58 ^a^	0.59 ^a^	0.65 ^b^	0.51 ^#§^	0.49 ^#§^	0.53 ^#§^
	SD	0.03	0.04	0.04	0.03	0.04	0.05	0.04	0.04	0.04

**Table 5:** Plasma isoflavone concentration in nmol/L of adult (DOP 4) DUC, DUhTP, and DU6 females that received diet A, S, or SPA. Statistical analysis was performed using two-way ANOVA. Significant differences between strains compared to the DUC and DUhTP lines are marked with # and ^§^. Significant differences between chow groups are marked with letters. For abbreviations, see Figure 1. Additional abbreviations: SD—standard derivation; G—glucuronide; S—sulfate.

Adult Females	Line	DUC			DUhTP			DU6		
	Diet Count	A n = 13	S n = 11	SPA n = 11	A n = 13	S n = 14	SPA n = 11	A n = 10	S n = 11	SPA n = 6
Total Isoflavone	mean	3541.7 ^a^	3075.2 ^a^	10.6 ^b^	3619.2 ^a^	2322.9 ^a^	4.5 ^b^	6738.4 ^#§a^	3448.4 ^b^	0.6 ^c^
	SD	875.1	1066.2	15.7	752.0	1165.4	6.3	2918.1	1259.6	1.0
Total Daidzein Equivalents	mean	846.6 ^a^	830.8 ^a^	4.6 ^b^	1342.1 ^#a^	703.5 ^b^	2.4 ^c^	1548.4 ^#a^	852.8 ^b^	0.0 ^c^
	SD	248.7	395.0	7.8	515.7	413.1	2.3	550.5	272.9	0.0
Daidzein (free Aglycone)	mean	119.6 ^a^	107.2 ^a^	0.0 ^b^	78.0 ^a^	36.6 ^a^	0.4 ^a^	316.0 ^#§a^	170.2 ^§b^	0.0 ^c^
	SD	46.4	63.0	0.0	37.0	18.6	1.4	127.1	63.2	0.0
Total Genistein Equivalents	mean	841.1 ^a^	997.0 ^a^	2.7 ^b^	1096.1 ^a^	655.6 ^a,b^	1.0 ^b^	2566.0 ^#§a^	966.2 ^b^	0.0 ^c^
	SD	226.1	479.6	4.9	354.3	390.3	2.3	1698.0	468.0	0.0
Genistein (free Aglycone)	mean	35.0 ^a^	35.4 ^a^	0.1 ^b^	24.3 ^a^	13.2 ^a^	0.5 ^a^	127.1 ^#§a^	55.8 ^§b^	0.3 ^c^
	SD	11.1	19.3	0.5	10.4	6.9	1.6	44.7	27.9	0.7
Sum of Equol, Equol-7-G, and Equol-4′-S	mean	1602.6 ^a^	1104.8 ^a^	3.2 ^b^	1078.6 ^a^	914.0 ^a^	0.2 ^b^	2181.2 ^§a^	1403.5 ^b^	0.4 ^c^
	SD	600.0	499.5	5.4	317.0	382.9	0.7	1068.4	794.7	0.9

## Data Availability

All raw data were generated at the FBN Dummerstorf and Max Rubner Institute in Karlsruhe. Derived data supporting the findings of this study are available on request from the corresponding author.

## Supplementary Materials

The following supporting information can be downloaded at: https://www.mdpi.com/article/10.3390/nu16162697/s1, Table S1: Limits of quantitation (LOQ) and limits of detection (LOD) for the analyses of isoflavones and their corresponding phase-II-metabolites in plasma using a UHPLC-MS/MS method. Table S2: Primer sequences for quantitative real-time PCR for selected genes. Table S3: Correlation studies between the total plasma IF concentration, the mRNA expression of Esr1 and Esr2, and the relative uterine and ovarian weights within the respective mouse lines DUC, DUhTP, DU6 (n = 10).
