# Supplementary material for: Impact of Dietary Isoflavones in Standard Chow on Reproductive Development in Juvenile and Adult Female Mice with Different Metabolic Phenotypes
Source: Nutrients. 2024 Aug 14;16(16):2697. doi: 10.3390/nu16162697 (PMC11357413; doi:10.3390/nu16162697)
Supplement: Supplementary file 1 [file nutrients-16-02697-s001.zip › nutrients-3113066-supplementary.pdf]

Supplementary material

Impact of Dietary Isoflavones in Standard Chow on Reproductive Development in Juvenile and Adult Female Mice with Different Metabolic Phenotypes

Authors: Zianka Meyer, Sebastian T. Soukup, Anna Lubs, Daniela Ohde, Christina Walz, Jennifer Schoen, Holger Willenberg, Andreas Hoeflich, Julia Brenmoehl

Table S1: Limits of quantitation (LOQ) and limits of detection (LOD) for the analyses of isoflavones and their corresponding phase-II-metabolites in plasma using a UHPLC-MS/MS method.

	Daidzein	Daidzein-4'-glucuronid	Daidzein-7-glucuronid	Daidzein-7,4'-diglucuronid	Daidzein-4'-sulfat	Daidzein-7-sulfat	Daidzein-7,4'-disulfat	Daidzein-7-glucuronid-4'-sulfat	Daidzein-4'-glucuronid-7-sulfat
LOQ (nmol/L)	3.6	18.8	14.8	15.2	20.4	8.8	45.6	5.6	3.6
LOD (nmol/L)	2.4	5.6	4.4	4.4	6.0	2.8	13.6	1.6	1.2

	Genistein	Genistein-4'-glucuronid	Genistein-7-glucuronid	Genistein-7,4'-diglucuronid	Genistein-4'-sulfat	Genistein-7-sulfat	Genistein-7,4'-disulfat	Genistein-7-glucuronid-4'-sulfat	Genistein-4'-glucuronid-7-sulfat
LOQ (nmol/L)	3.2	21.6	12.8	21.6	18.0	11.2	23.6	8.0	6.0
LOD (nmol/L)	0.8	6.4	4.0	6.4	5.2	3.6	7.2	2.4	1.6

	Equol	Equol-7-glucuronid	Equol-4'-sulfat
LOQ (nmol/L)	560.8	60.8	4.4
LOD (nmol/L)	168.0	18.0	1.2

Table S2: Primer sequences for quantitative real-time PCR for selected genes

Gene	Sequence 5' → 3'		Amplification efficiency	Gene Bank Accession
Actb	forward	TGACAGGATGCAGAAGGAGA	1.97	NM_007393.5
	reverse	CGCTCAGGAGGAGCAATG		
Sdha	forward	CAAATTCTCTCTTGGACCTTGTAGT	1,88	NM_023281.1
	reverse	CCTTAATTGAAGGAACTTTATCTCCA		
Gapdh	forward	GGCTCCCTAGGCCCCTCCTG	2.01	NM_001289726.1
	reverse	TCCCAACTCGGCCCCCAACA		
Esr1	forward	CCCGCCTTCTACAGGTCTAAT	1.98	NM_007956.5
	reverse	CTTTCTCGTTACTGCTGGACAG		
Esr2	forward	CTGTGATGAACTACAGTGTTCCC	1.94	NM_207707.1
	reverse	CACATTTGGGCTTGCAGTCTG		

Abbreviations: Actb – actin beta, Sdha – succinate dehydrogenase flavoprotein subunit A, Gapdh - glyceraldehyde-3-phosphate dehydrogenase, Esr1/2 - estrogen receptor ½

Table S3: Correlation studies between the total plasma IF concentration, the mRNA expression of Esr1 and Esr2, and the relative uteri and ovary weight within the respective mouse lines DUC, DUhTP, DU6 (n = 10).

Correlation		DUC	DUhTP	DU6
total IF / Esr1 expression	r	0.333	0.053	-0.253
	p	ns	ns	ns
total IF / Esr2 expression	r	0.049	0.458	**-0.666**
	p	ns	ns	*
total IF / rel. uteri weight	r	0.361	-0.250	0.075
	p	ns	ns	ns
total IF / rel. ovary weight	r	0.5917	0.524	-0.376
	p	ns	ns	ns

Significant correlations were calculated by Pearson correlation, are expressed in bold letters, and indicated with two-tailed p-value * $p < 0.05$. r, Pearson correlation coeficient; ns, not significant; Esr1/2, estrogen receptor 1/2
